# Function of different proportions of apolipoprotein A-I cysteine mutants and apolipoprotein A-V on recombinant high-density lipoproteins *in vitro*

**DOI:** 10.1042/BSR20190547

**Published:** 2019-05-17

**Authors:** Ying Yan, Shulai Lu, Shaoyou Jia, Qingzhe Dong, Lei Wang, Yunlong Wang

**Affiliations:** 1 Biological Specimen Bank, The Affiliated Hospital of Qingdao University, Qingdao 266000, China; 2 Department of Pharmacology, School of Pharmacy, Qingdao University, Qingdao 266021, China; 3 Stomatological Department, Qingdao Municipal Hospital, Qingdao 266021, China; 4 The Affiliated Hospital of Qingdao University, Qingdao 266000, China

**Keywords:** apoA-I, apoA-I Milano, apoA-I (N74C), apoA-V, rHDLs

## Abstract

To explore the anti-atherosclerotic effects of recombinant high-density lipoproteins (rHDL) of apolipoprotein AI wild-type (apoA-Iwt), apolipoprotein AI Milano (apoA-IM), apolipoprotein AI (N74C) (apoA-I (N74C) )and apolipoprotein AV (apoA-V). We constructed rHDL liposomes (rHDLs), which included apoA-Iwt, apoA-IM, and apoA-I (N74C), followed by the synthesis of rHDLs, with the indicated ratios of apoA-Iwt, apoA-IM, apoA-I (N74C) and apoA-V. We investigated the anti-atherosclerotic effects by experiments including the DMPC clearance assay and experiments that assessed the *in vitro* antioxidation against low-density lipoprotein, the cellular uptake of oxidized low-density lipoprotein (oxLDL) and the *in vitro* intracellular lipid accumulation. Electron microscopy results revealed that as more apoA-V was present in rHDLs, the particle size of rHDLs was larger. The DMPC clearance assay subsequently showed that rHDL protein mixtures could promote DMPC turbidity clearance when more apoA-V was included in the reaction mixtures, with apoAV-rHDL showing the strongest turbidity clearance ability (*P<*0.05 vs AI-rHDL). *In vitro* antioxidation against low-density lipoprotein assays indicated that rHDLs containing apoA-V had increasing oxidation resistance against low-density lipoprotein (LDL) with higher apoA-V contents. Finally, cellular uptake of oxLDL and intracellular lipids suggested an apparent oxidation resistance to LDL oxidation *in vitro* and a reduced intracellular lipid accumulation in THP-1-derived macrophages, with AIM-rHDL demonstrating the greatest ability to decrease intracellular lipid accumulation. Different proportions of apolipoprotein A-I cysteine mutants and apolipoprotein A-V of rHDL changed the lipid binding capacity, particle size, and antioxidant capacity. These changes may show a beneficial effect of rHDL on atherosclerosis.

## Introduction

Recently, an increasing number of studies have focussed on atherosclerosis (AS), which is caused by the accumulation of plaque in the lining of arteries, resulting in cardiovascular disease, cerebral infarction, and peripheral vascular disease. Coronary Heart Disease (CAD) is the main cause of death worldwide, leading to approximately 30% of the annual global mortality. [1] Some studies have shown that a high HDL level can clearly decrease the risk of AS. HDL proteins not only mainly act on reverse cholesterol transport (RCT) but also have many other helpful biological functions, including antioxidative, anti-inflammatory, vasodilatory, antithrombotic, and cytoprotective effects [2–5].

In addition, ApoA-Iwt constitutes approximately 70% of HDL proteins [6], which plays an important role in RCT, lowering the level of triglycerides. Mature ApoA-Iwt is a single polypeptide chain composed of 243 amino acids, and more than 50% of its structure consists of an α helix. ApoA-Iwt is an apolipoprotein with multiple biological functions and the major function is to stimulate RCT, which plays a key role in delivering cholesterol from peripheral tissues – such as macrophages and foam cells in atherosclerotic lesions – to the liver. Two mutations of ApoA-I have been reported, namely, ApoA-I Milano (apoA-IM) and ApoA-I Paris, in which ApoA-IM shows distinct effects of very low high-density lipoprotein cholesterol (HDL-C) levels and moderate hypertriglyceridemia but without evidence of premature CAD, preclinical coronary or carotid atherosclerosis [7,8]. A study in which the researchers replaced the mouse apoA-Iwt gene with the human apoA-IM mutation showed that mice expressing ApoA-IM had low concentrations of the human apolipoprotein and reduced HDL-C levels [9]. On account of the Edmundson wheel [10], we designed seven cysteine mutants of apoA-Iwt, one of which, named apoA-I (N74C), showed similar antiatherogenic properties to apoA-IM based on previous scientific research.

Furthermore, apoA-V was revealed when genomic DNA sequences were compared between humans and mice, and, similar to apoA-Iwt, it is located proximal to the APOAI/CIII/AIV gene cluster on human 11q23 [11]. The apolipoprotein gene cluster (APOAI/CIII/AIV) is an essential component affecting lipid parameters in plasma [12]. Studies investigating apoA-V have noted an obvious influence on plasma triglyceride levels [13] as well as an increase in apoA-I (N74C) levels in HDL and even an increase in the number and size of HDL particles at higher concentrations [14,15]. The neighboring position of apoA-Iwt and apoA-V in the same gene cluster and the modulation of apoA-V on HDL might indicate a unique relationship between apoA-V and HDL [16].

Taken together, these findings indicate that apoA-Iwt or its cysteine mutations and apoA-V exhibit inhibitory effects on AS; one influences plasma cholesterol levels and the other affects plasma triglyceride levels. Therefore, we synthesized a series of rHDLs with the indicated ratio of apoA-Iwt/ApoA-IM/apoA-I (N74C) and apoA-V to investigate the cooperative effect in AS.

## Materials and methods

### Materials

The 1, 2-dipalmitoyl-sn-glycero-3-phosphocholine (DPPC) and the PMSF were purchased from Sigma–Aldrich (St. Louis, MO, U.S.A.); the Pierce BCA Protein Assay kit was purchased from Thermo Fisher Scientific (Rockford, IL, U.S.A.). Ni-NTA His Bind resin and ultrafiltration centrifugal tube were purchased from Millipore (Billerica, MA, U.S.A.); RPMI-1640 and Certified were purchased from Biological Industries (Kibbutz Beit Haemek, Israel). Human THP-1 monocytes and recombinant Escherichia coli containing the coding region for the human ApoA-Iwt and cysteine mutants were preserved in the Laboratory of The Affiliated Hospital of Qingdao University. All other chemical reagents were obtained commercially and were of analytical grade.

### Preparation of recombinant apoA-Is and apoA-V

The expression and purification of recombinant ApoA-Iwt and cysteine mutants were performed as previously described [17,18]. The recombinant apoA-Iwt and its cysteine mutants were purified by nickel column chromatography and concentrated using enrichment centrifuge tubes. Purified proteins were identified by SDS/PAGE (12%) and stored at −80°C for use in subsequent assays.

### Synthesis of recombinant HDL

We synthesized recombinant HDL using the sodium cholate dialysis technique. The recombinant HDL was in suitable mass ratios of DPPC to apolipoproteins of 3.35:1. More specifically, DPPC was dissolved in 725 mM sodium cholate solution in Tris-HCl buffer (10 mM Tris-HCl, 150 mM NaCl, 1 mM EDTA, pH 7.4), and then the dissolved DPPC was mixed with the recombinant apolipoproteins at various mass ratios of apoA-Iwt/apoA-IM/apoA-I (N74C):apoA-V of 1:0, 100:1, 1:1, 1:100 or 0:1. The mixtures were subjected to ultrasonication in an ice bath, and the optimum was obtained at 100 W, 10 min. To remove cholate, it is normal to perform dialysis for 20 h against saline at 4°C. The rHDLs were concentrated by ultrafiltration, and the recombinant HDLs were stored at 4°C.

### Identification of recombinant HDLs

The recombinant HDLs were identified by electron microscopy.

### DMPC turbidity clearance assay

To evaluate the capacity of various rHDL protein mixtures to bind lipids, the ability to clear the lipid turbidity was determined based on the value rate constant k1/2. The interaction of the rHDL mixtures with DMPC was monitored as previously described [18].

### Antioxidant assay against LDL *in vitro*

All constructed rHDLs were tested for their antioxidant activity against LDL-peroxidation. To examine the anti-LDL oxidation of rHDLs, we used the copper-mediated LDL oxidation system and the TBARS assay to verify the extent of oxidation of LDL *in vitro*. The formation of products from the peroxidation of LDL had maximum absorption peaks at 532 nm, and three independent readings were performed at 532 nm.

### Intracellular lipid accumulation

To analyze the different influences of recombinant HDLs on the decrease in intracellular lipid accumulation, we studied the intracellular lipid accumulation modulated by rHDLs using differentiated human THP-1 macrophages as a cell model.

### Statistical analysis

Values are shown as the mean ± S.D., and differences between multiple groups were analyzed by one-way ANOVA using SPSS 21.0 software for Windows (SPSS Inc., U.S.A.), the differences between two groups were determined by Student’s *t* test. The *P*<0.05 was considered statistically significant. Graph construction was performed with Prism 6.0 (GraphPad Software Inc.).

## Results

### Purification and identification of rHDLs

**Figure 1:**
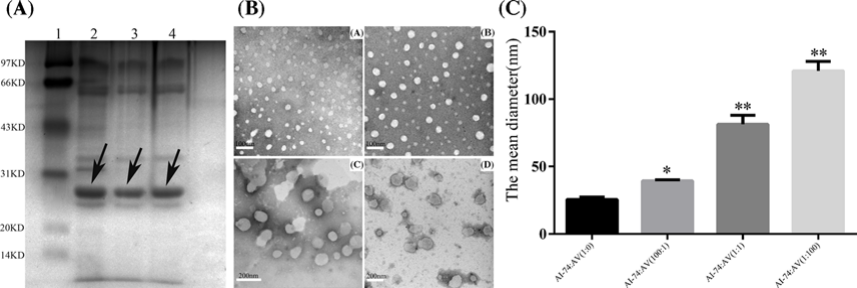
Purification and identification of rHDLs **(A)** Recombinant purified ApoA-Iwt and its mutants were examined by SDS/PAGE (12% gel). Protein molecular weights of the wild-type ApoA-Iwt, apoA-IM and apoA-I (N74C) both were 29 kDa. Lanes 1–4 show the low molecular mass marker, wild-type ApoA-I, apoA-IM, and apoA-I (N74C). **(B)** The size and morphology of rHDLs reconstituted with apoA-I (N74C) and apoA-V. Electron micrograph of the negatively stained rHDLs observed under a transmission electron microscope. (A) AI-74-rHDL; (B) AI-74:AV(100:1)-rHDL; (C) AI-74:AV (1:1)-rHDL; (D) AI-74:AV(1:100)-rHDL. **(C)** The mean diameter of rHDLs reconstituted with apoA-I (N74C) and apoA-V. *P< 0.05, **P< 0.01 for comparison with AI-rHDL.

The purification of proteins was performed by SDS/PAGE (12% protein gel), and the 28-kDa protein in the lysate was detected by Coomassie bright blue staining (Figure 1A). Then, thirteen rHDLs were assessed, including apoA-Iwt-rHDL (AI-rHDL), apoA-IM-rHDL (AIM-rHDL), apoA-I (N74C)-rHDL (AI-74-rHDL), apoA-V-rHDL (AV-rHDL), apoA-Iwt:apoA-V(100:1)-rHDL (AI:AV(100:1)-rHDL), apoA-IM:apoA-V(100:1)-rHDL (AIM:AV(100:1)-rHDL), apoA-I (N74C):apoA-V(100:1)-rHDL (AI-74:AV(100:1)-rHDL), apoAIwt-:apoA-V(1:1)-rHDL (AI:AV(1:1)-rHDL), apoA-IM:apoA-V(1:1)-rHDL (AIM:AV(1:1)-rHDL), apoA-I (N74C):apoA-V(1:1)-rHDL (AI-74:AV(1:1)-rHDL), apoA-Iwt:apoA-V(1:100)-rHDL (AI:AV(1:100)-rHDL), apoA-IM:apoA-V(1:100)-rHDL (AIM:AV(1:100)-rHDL), and apoA-I (N74C):apoA-V(100:1)-rHDL (AI-74:AV(1:100)-rHDL). The original mass ratio of DPPC: apolipoproteins was 3.35:1 and the rHDLs were identified by electron microscopy. We measured the shape and size of rHDLs in the apoA-I (N74C) group by staining with 1% sodium phosphotungstate, pH 7.4, as previously described [19] (Figure 1B). The results (Figure 1C) showed that the addition of apoA-V to rHDLs could result in the formation of larger particles.

### DMPC turbidity clearance assay

**Figure 2:**
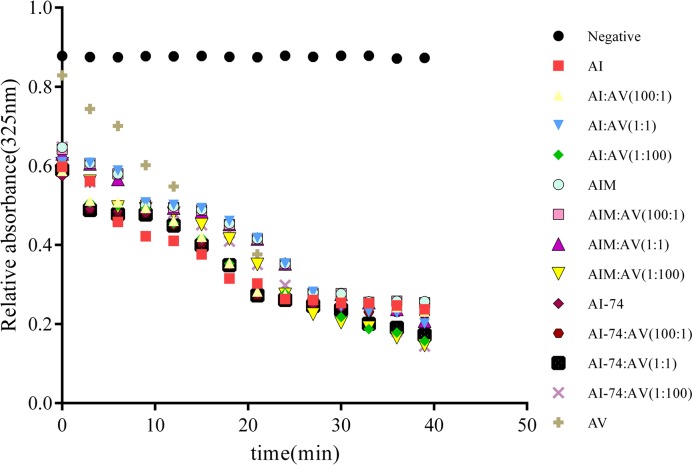
Kinetics of the interaction of the described protein mixtures of apoA-Is and apoA-V with DMPC multilamellar liposomes The changes in turbidity were monitored by the change in absorbance at 325 nm at 3 min intervals for the initial 40 min and plotted as a function of time.

The DMPC turbidity clearance assay was used to measure the abilities of rHDL protein mixtures to combine with lipids. The level of ability was represented by K1/2, which was higher in the AI-74:AV(1:100)-rHDL group; the other groups had lower values relative to the AIM-rHDL group (Table 1). These data suggested that rHDLs could promote the clearance of DMPC turbidity when more apoA-V was included in the reaction mixtures (Figure 2). Compared with the three groups with the same proportions, the apoA-I (N74C) group showed stronger ability to clear turbidity, that is, stronger lipid-binding ability. In each group, with the increase in the A-V ratio in rHDLs, the K values of rHDLs increased, which indicated a stronger lipid-binding ability. These results suggested that apoA-V had a higher lipid-binding ability than apoA-Iwt. The increase in apoA-V content in rHDL can allow rHDL to bind more phospholipids.

**Table 1:** Rate constants for the decrease in turbidity after mixing the described protein mixtures of apoA-Is and apoA-V with DMPC

Groups	K (min-1)
AI-rHDL	0.02371 ± 0.010
AI:AV(100:1)-rHDL	0.02384 ± 0.018
AI:AV(1:1)-rHDL	0.02837 ± 0.010*
AI:AV(1:100)-rHDL	0.03445 ± 0.010*
AIM-rHDL	0.02369 ± 0.002*
AIM:AV(100:1)-rHDL	0.02376 ± 0.007▲
AIM:AV(1:1)-rHDL	0.02856 ± 0.010▲
AIM:AV(1:100)-rHDL	0.03656 ± 0.010▲
AI-74-rHDL	0.02420 ± 0.010*
AI-74:AV(100:1)-rHDL	0.02480 ± 0.005
AI-74:AV(1:1)-rHDL	0.03158 ± 0.002•
AI-74:AV(1:100)-rHDL	0.03853 ± 0.030•
AV-rHDL	0.04133 ± 0.010*

(K denotes the rate of the decrease in turbidity). (Each value represents the mean ± S.D. of at least three independent determinations. *P< 0.05 vs AI-rHDL, *P<* 0.05 vs AIM-rHDL, *P<* 0.05 vs AI-74-rHDL).

### Antioxidant assay against LDL *in vitro*

**Figure 3:**
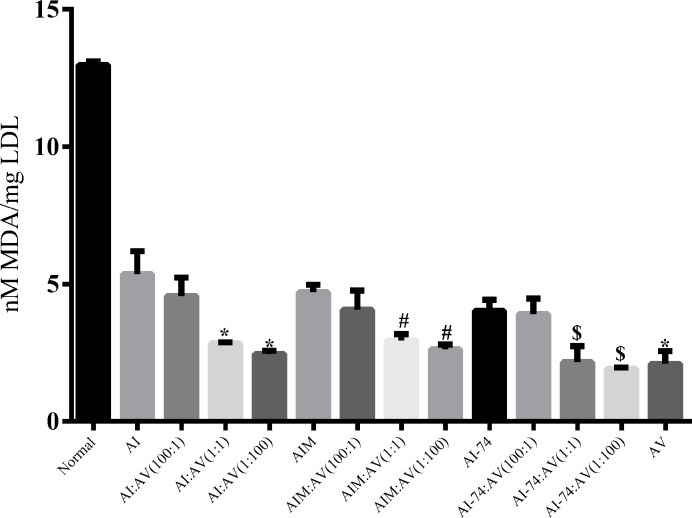
Antioxidant activity of rHDLs reconstituted with apoA-Iwt /apoA-IM/apoA-I (N74C) and apoA-V via the cholate dialysis method *in vitro* (Each value represents the mean ± S.D. of at least three independent determinations. *P<0.05 vs AI-rHDL, #*P<*0.05 vs AIM-rHDL, $*P<*0.05 vs AI-74-rHDL).

To assess the antioxidant ability of the rHDLs, we used the LDL oxidation reaction-mediated Cu2^+^ and TBARS system to measure the degree of LDL oxidation *in vitro*. The formation of MDA was detected by the TBARS system, which can reflect the extent of oxidation of the LDL in different groups. Compared with the negative group, the rHDLs displayed antioxidant activity, particularly in the AI-74:A-V(1:100)-rHDL, which was significantly more potent, with an MDA of 1.917 nM/mg LDL. In the apoA-Iwt group, A-I:A-V(1:1)-rHDL and A-I:A-V(1:100)-rHDL decreased significantly to a value lower than the MDA levels of the A-I-rHDL and A-I:A-V(100:1)-rHDL groups. However, the apoA-IM groups demonstrated nearly equal power to resist oxidation compared with the apoA-Iwt group. In addition, the apoA-I (N74C) showed the strongest antioxidation effect compared with apoA-Iwt and apoA-IM. Moreover, the rHDLs containing apoA-V displayed an increasing oxidation resistance against LDL with an increasing apoA-V content (Figure 3).

### Intracellular lipid accumulation

**Figure 4:**
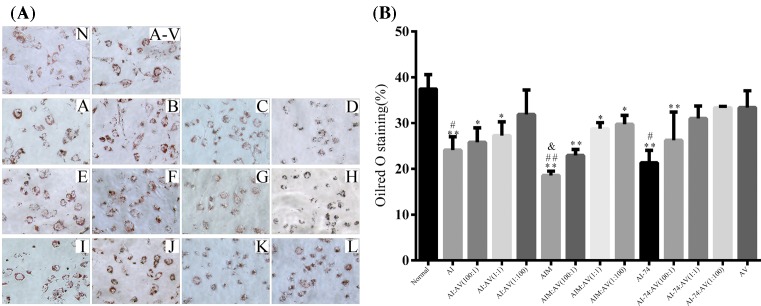
THP-1-derived macrophages stained by oil-red O **(A)** Intracellular lipid accumulation in the presence of rHDLs in THP-I-derived macrophages. The area of oil-red O was equal to (N): images of oil-red O staining in THP-1-derived macrophages at 20× magnification. PBS-treated; (A-V): A-V; (A-L): AI, AI:AV(100:1), AI:AV(1:1), AI:AV(1:100), AIM, AIM:AV(100:1), AIM:AV(1:1), AIM:AV(1:100), AI-74(1:0), AI-74:AV(100:1), AI-74:AV(1:1), AI-74:AV(1:100). **(B)** Quantitative analysis of the intracellular lipid accumulation in THP-1-derived macrophages. The areas of oil-red O staining were calculated using Image Proplus software for at least 15 cells per sample. (Each value represents the mean ± S.D. of at least three independent determinations. *P<0.05, **P<0.01 vs normal, #*P<*0.05, ##*P<*0.01 vs AI-rHDL, &*P<*0.05 vs AV-rHDL).

**Figure 5:**
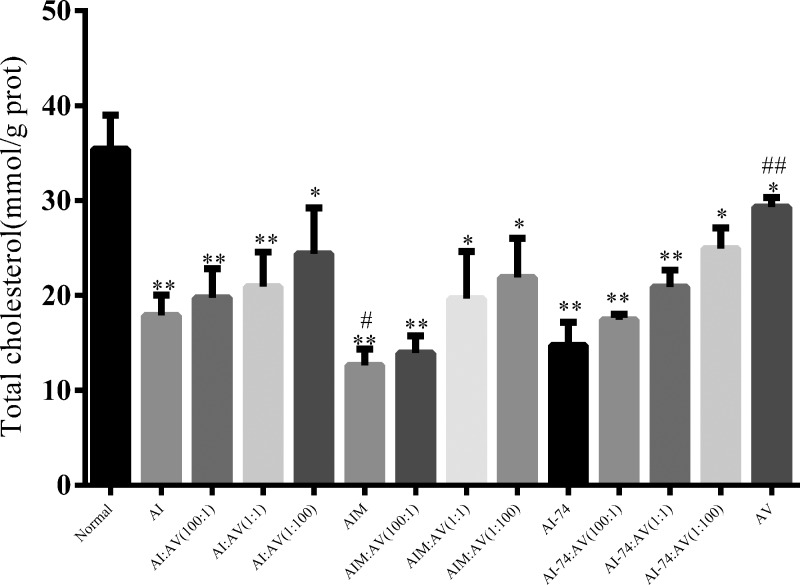
To verify the data for the lipid staining area, the intracellular total cholesterol level expressed as the ratio of total cholesterol to total protein was analyzed (Each value represents the mean ± S.D. of at least three independent determinations. *P< 0.05, **P< 0.01 vs normal, #*P<*0.05, ##*P<* 0.01 vs AI-rHDL).

To analyze the influence of rHDLs on the ability to promote intracellular lipid accumulation, we used human THP-1 cells differentiated into macrophages as a cell model to assess intracellular lipid accumulation ability. OxLDL-loaded THP-1 cells derived from foam cells were stained with oil-red O and examined using Image Proplus software (Figure 4A). Compared with the untreated group, the results revealed a significant reduction in foam cell formation after treatment with rHDLs. Compared with the apoA-Iwt groups and the apoA-I (N74C) groups in equal proportions, the apoA-IM groups exhibited stronger anti-intracellular lipid accumulation ability in THP-1 macrophages (Figure 4B). Figure 5 shows at the same ratio in the apoA-IM groups showed the smallest area of oil-red staining; apoA-I (N74C) showed a smaller area stained by oil-red, which illustrated that apoA-IM and apoA-I (N74C) inhibited the cellular uptake of oxLDL. In all four groups, rHDLs with more apoA-Iwt or its cysteine mutant component showed stronger inhibition of the uptake of oxLDL that was not associated with the apoA-V component. We also used the total cholesterol (TC) assay kit to determine intracellular TC levels in each group to verify the oil-red O staining results (Figure 5). Figure 5 shows consistent results with those shown in Figure 4B.

## Discussion

In the present study, we constructed 13 recombinant HDLs (apoA-Iwt-rHDL, apoA-IM-rHDL, apoA-I (N74C)-rHDL, apoA-V-rHDL, apoA-Iwt:apoA-V(100:1)-rHDL, apoA-IM:apoA-V(100:1)-rHDL, apoA-I (N74C):apoA-V(100:1)-rHDL, apoAIwt-:apoA-V(1:1)-rHDL, apoA-IM:apoA-V(1:1)-rHDL, apoA-I (N74C):apoA-V(1:1)-rHDL, apoA-Iwt:apoA-V(1:100)-rHDL, apoA-IM:apoA-V(1:100)-rHDL, and apoA-I (N74C):apoA-V(100:1)-rHDL) by mixing DPPC with certain percentages of apoA-Iwt/apoA-IM/apoA-I (N74C) and apoA-V. Next, we examined the *in vitro* effects of different rHDLs using several experiments.

The results from the DMPC turbidity clearance assay showed that in each group, larger K values were obtained when more apoA-V was added to the rHDL mixtures, with the apoA-V group demonstrating the highest K value (*P<*0.05 vs AI-rHDL). Thus, stronger lipid binding capacity was achieved with greater apoA-V content in rHDL mixtures, which indicated that apoA-V had a stronger binding capacity for lipid than apoA-Iwt.

The initial event in atherogenesis is the endodermal retention and oxidative modification of LDL [20]. *In vitro* assays have indicated that oxidative modification of LDL can be altered by copper ions, resulting in increased absorption of lipoprotein into macrophages [21,22]. Previous studies have shown that increasing the apoA-Is content in rHDLs strengthens the antioxidative activity toward oxLDL via ABCA1 [23], whereas little is known about apoA-V or about the combination of apoA-Is and apoA-V. In our research, we applied the copper-mediated LDL oxidation system to measure the antioxidative effects, and the results suggested that rHDLs had antioxidative activity *in vitro*, which increased with the addition of apoA-V to the rHDLs. This phenomenon might be related to the antioxidation function of apoA-Is/apoA-V itself or the interaction between apoA-V and phospholipids. However, the underlying mechanism and clinical significance require further investigation.

The crucial period of the physiological course of atherosclerosis occurs when, upon adhesion to the endothelium, monocytes migrate into the inner membrane where they differentiate into macrophages, accumulate lipids, and become foam cells [24]. LDL oxidation is the first event in the formation of foam cells [25,26], in which LDL lipids in human arterial lesions are widely oxidized and oxLDL is evident *in vivo* [27]. We chose the oxLDL-challenged THP-1 macrophage model as an *in vitro* model to measure the degree of lipid or oxLDL uptake into cells, which occurred in the presence of each rHDL and demonstrated that AI/AIM/AI-74(alone)-rHDL significantly inhibited the uptake of oxLDL and the accumulation of cellular lipids, while the addition of apoA-V to rHDLs resulted in increased cellular lipid accumulation. This finding is consistent with previously reported results showing that apoA-V may competitively inhibit apolipoprotein- or HDL-mediated efflux of cholesterol from cells at higher concentrations [28].

In summary, the data presented herein provide some evidence for an intracellular mode of action of rHDLs. The rHDLs showed increases in lipid-binding ability, antioxidative capacity, and particle size with the addition of more apoA-V, although apoA-V showed no association with the cellular uptake of oxLDL and intracellular lipid accumulation in THP-1-derived macrophages. However, apoA-IM and apoAI-74 both showed better effects than apoA-Iwt (*P<*0.05, vs AI-rHDL). Better lipid-binding ability, stronger antioxidative capacity and less intracellular lipid accumulation indicates stronger anti-arteriosclerosis ability. Nevertheless, the present studies are far from sufficient, and the underlying mechanism and future clinical applications require further elucidation.

## Abbreviations

apoA-IM: apolipoprotein AI Milano
apoA-Iwt: apolipoprotein AI wild-type
apoA-I(N74C): apolipoprotein AI (N74C)
AS: atherosclerosis
CAD: coronary heart disease
DMPC: 1,2-dimyristoyl-sn-glycero-3-phosphocholine
DPPC: 1,2-dipalmitoyl-sn-glycero-3-phosphocholine
HDL-C: high-density lipoprotein cholesterol
LDL: low-density lipoprotein
oxLDL: oxidized low-density lipoprotein
RCT: reverse cholesterol transport
rHDL: recombinant high-density lipoprotein
TC: total cholesterol
